# Evaluation of a new mouthwash on caseous formation

**DOI:** 10.1016/S1808-8694(15)30752-7

**Published:** 2015-10-19

**Authors:** Maurício Duarte da Conceição, Luciana Sassa Marocchio, Olinda Tárzia

**Affiliations:** 1 Former President of the Brazilian Association of Mouth Odors Studies and Research, Graduate Degree in Halitosis – Centro de pós-graduação da Faculdade de Odontologia São Leopoldo Mandic; 2 MSc in Oral Pathology – Faculdade de Odontologia da Universidade de São Paulo – Bauru, Graduate Degree in Halitosis – Centro de pós-graduação da Faculdade de Odontologia São Leopoldo Mandic; 3 PhD in Oral Biochemistry – Faculdade de Odontologia da Universidade de São Paulo – Bauru. Coordinator of the Graduate Program in Halitosis – Centro de pós-graduação da Faculdade de Odontologia São Leopoldo Mandic

**Keywords:** mouthwashes, sulfur compounds, halitosis, tongue, double-blind method, tonsils

## Abstract

Tonsil caseous affects a significant percentage of the population. Surgeries, conservative or not, have been the only viable alternatives of treatment. However, today there is still not, up to now, an economical and non-invasive treatment that presents satisfactory results.

**Aims:**

The objectives of this study are to evaluate the efficiency of a mouthwash, with active ingredients that associate oxygenating and antimicrobial substances, in the reduction of caseous and tongue coating formation, whose etiology is similar to caseous, and to evaluate the reduction of the volatile sulfur compounds (VSCs) concentration.

**Study design:**

Double blind, placebo-controlled, randomized, clinical and experimental study.

**Material e methods:**

A sample of 50 volunteers with more than one year of chronic caseous tonsillitis complaint used it. The research was carried out in 2005, in the cities of São Paulo and Campinas.

**Results:**

For the group that used the placebo solution, there was no correlation between the variables or statistical significance in the results. For the group that used the mouthwash, the results were significant in all analyzed questions.

**Conclusions:**

This new mouthwash proved to be a viable conservative alternative for the treatment of tonsil caseous, being also efficient in the reduction of tongue coating formation and VSCs concentration.

## INTRODUCTION

**Figure 1:**
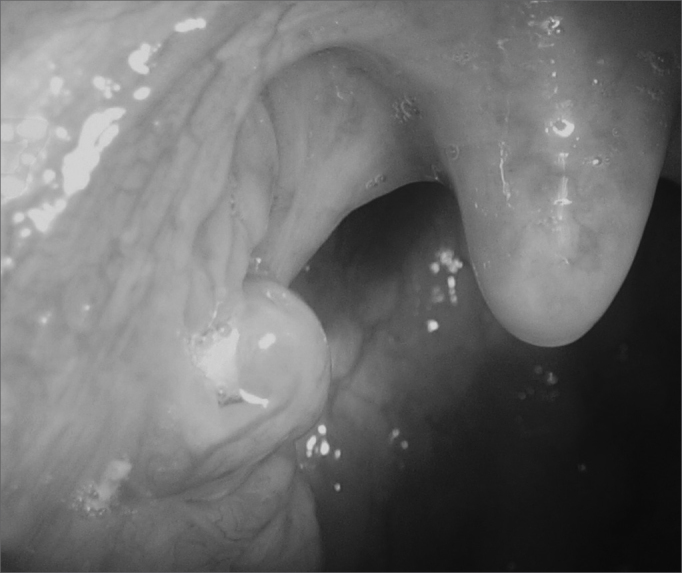
Caseum inside a tonsil crypt.

Caseum can build up in the pharyngeal tonsils' cavities or crypts (Figure 1), basically, it happens when there is salivary flow reduction or epithelial shedding above the physiological limits, or both occurring simultaneously^1^. It is a yellowish viscous and smelly mass, than can be expelled during speech, cough or sneezes. Its name derives from the Latin word “caseum”, which means cheese, because it is similar to a small “cheese ball”.

It is made up of epithelial cells that shed off the oral mucosa, salivary proteins and protein remains of food, that will serve as food stuff to the proteolytic anaerobe bacteria that dwell there.^1^ Bacterial metabolism produce a foul smelling compound at the end, called Volatile Sulphur Compounds (VSC) – sulfur-derived gases. These gases, as they reach a certain concentration, give the breath a characteristic smell of sulfur or that of a rotten egg, making up a common etiology for chronic halitosis^2^^,^^3^.

Tonsillitis or caseous chronic tonsillitis have its signs and symptoms reported as a feeling of frequent throat discomfort or irritation, and is characterized by shedding tonsil caseum alone or associated with other symptoms such as halitosis, feeling of foreign body or repetition tonsillitis. Such symptoms may still be followed of hyperemia and pharyngeal tonsil hypertrophy, without fever, and usually disappear after the caseum is shed. This disorder can happen at any age, especially in individuals who have never had tonsil symptoms^4^.

Controlling tonsil caseum is an important tool to prevent diseases, because the bacteria present in this process (practically the same in the tongue saburra) are associated with the etiology of some diseases such as gastritis, pneumonia and periodontal disease, the latter is also associated with many other systemic disorders^1^.

The clinical treatment^5^ proposed for tonsil caseum so far involve the use of anti-inflammatory agents, gargling substances with saline and antiseptic solutions, without satisfactory results; or the surgical treatment by means of radical or conventional tonsillectomies^6^^,^^7^, or even laser-assisted surgery^8^, ^9^, ^10^, ^11^. The valid surgical option to treat tonsil caseum has been laser-assisted cryptolisis^4^^,^^12^, which is based on removing parts of the crypts, though preserving part of the pharyngeal tonsils. The problems this method presents are, depending on the technique used, due to volatilization or coagulation, a painful postoperative recovery, the fact that many surgical interventions may be required and its cost, which is still high, especially in Brazil.

Recognizing pharyngeal tonsils as a lymphoepithelial organ with an important immune function^13^, ^14^, ^15^ and for that, in principle, must be preserved, led us to try and find a conservative method to inhibit the formation of caseum and, consequently, keep the pharyngeal tonsils. The literature findings on mouth rinsing substances in the prevention of caseum formation are insignificant. An inverse situation happens with tongue saburra, which has considerable literature on which substances have a positive effect on its formation^1^^,^^16^^,^^17^. Tongue saburra is made up just like caseum1. Thus, hypothetically the substances that act on the lingual saburra could have a positive effect, also on tonsil caseum. Therefore, the use of a mouthwash, which is the goal of the present investigation, specifically developed for this purpose, would mean an important option for conservative treatment of tonsil caseum.

This new mouthwash was patented by the Halitus® company, together with Propeq, a junior company from the school of chemical engineering of the State University of Campinas – Unicamp, taking into account the legislation in effect from the National Agency of Sanitary Surveillance (ANVISA), as well as all the recommendations from the Food and Drug Administration (FDA) and the many pharmacopoeias from many countries.

The goals of the present investigation are to assess the efficacy of this new mouthwash in reducing the formation of tonsil caseum and tongue saburra – with similar etiology to that of the caseum; and to asses the reduction in volatile sulphur compounds (VSCs) concentration.

## MATERIALS AND METHODS

The study was designed o be a contemporary longitudinal cohort; clinical and experimental, randomized and double blind, placebo controlled.

This study involved the participation of human beings, and it was approved under protocol number 05/291, issued on July 20, 2005 by the Ethics in Research Committee of the School of Dentistry of São Leopoldo/Mandic.

The voluntaries were submitted to screening and selected based on the following inclusion criteria: a complaint of chronic caseous tonsillitis for more than one year, having caseous shedding as the main symptom and caseous shedding alone or followed by other symptoms such as halitosis, a feeling of foreign body and/or acute tonsillitis. We used a sample made of healthy adults, of any gender, older than 18 years of life and non-users of illicit drugs.

Each volunteer was assessed three times: initial evaluation, after four weeks and final evaluation − 8 weeks after the study began, in three items:

1) Frequency of shedding caseum;
2) Halitometry – in order to check for VSC in parts per billion (PPB) by making use of a Halimeter® RH 17;
3) Tongue saburra volume (a 0 to 5 scale, advocated by the author):

0- No saburra;
1- Mild saburra present in the tongue's posterior third;
2- Mild saburra present in the medium and posterior thirds of the tongue;
3- Moderate saburra in the posterior third of the tongue;
4- Moderate saburra in the medium and posterior thirds of the tongue;
5- Moderate saburra in the anterior, medium and posterior thirds of the tongue.

Observation: a) Mild saburra: the lingual papillas are visible, despite the tongue saburra; b) Moderate saburra: lingual papillas are not visible, and are covered by the tongue saburra.

The volunteers were informed of the objective of this study and signed an informed consent form.

The volunteers were called by the publication of notes about the research in popular newspapers of large circulation. We needed two adds to reach a total of 62 volunteers. Of these, 31 used flasks with the placebo solution and 31 used the mouthwash.

### Composition

Placebo: demineralized water, dye, essence and edulcorant;

Mouthwash: demineralized water, dye, and essence, edulcorant, fluorine, preservative, solubilizer, hydrogen peroxide, sodium perborate and cetylpyridinium chloride.

The different solutions were randomly given to the volunteers, by order of service in the initial evaluation, with the letters A and B for the different groups, for a first group of volunteers, and letters C and D for the second group. This second group joined the study some weeks after the first group, because it was difficult to find volunteers after only one add campaign.

Mode of use: the subjects were asked to gargle 5 ml of the solution for 20 seconds every 12 hours (morning and night), followed by mouthing the same solution for 20 seconds. After that the volunteers were instructed to spit the entire solution and not to drink, eat or wash their mouths for at least 20 minutes. Moreover, each volunteer was instructed as to keep using the product, not miss any of the sessions and keep the same oral hygiene routine they had been following before the study so as to prevent any alteration from impacting the frequency at which caseum appeared in the oral cavity, VSC concentration and tongue saburra quantity.

At the end of the study, 12 of these volunteers were excluded for the following reasons: they abandoned the study or did not come to one of the sessions for personal reasons or on account of disease, and also for having temporarily interrupted the use of the product.

The average volunteer age as 40 years. Users of fixed, partial and removable dental prosthesis were 24%; and 4% wore orthodontic braces. Their gums were considered good in 70% of the cases; while 30% had gingivitis; 20% had mild gingivitis, sporadic gum bleeding when hey flossed and 10% with moderate gingivitis, with gum bleeding at least twice a week, with dental floss and /or tooth brush.

The information about caseum shedding, as well as VSC readings and tongue saburra levels were noted in the volunteers' charts and they also signed the charts in order to authenticate the truthfulness of the information collected.

Their tongues were photographed in each session. Using a 5.1 Mega pixels Nikon Coolpix 5200 digital camera, for comparative analysis of the tongue appearances.

**Figure 2:**
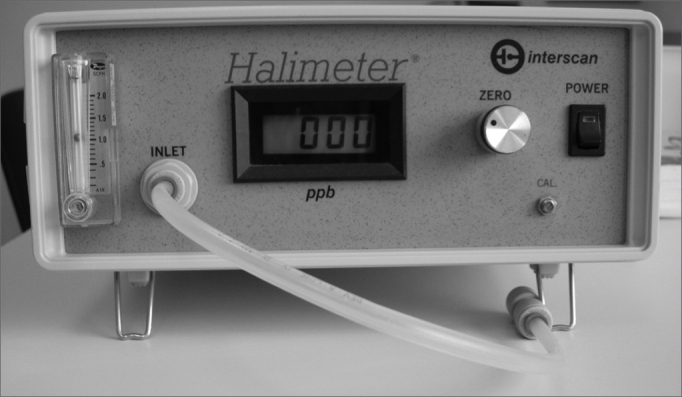
Halimeter® RH 17.

Halitometry – to check for VSC concentrations – in parts per billion (ppb) – we used a Halimeter® RH 17, for these measures (Figure 2).

## RESULTS

The average results from the group that used the placebo solution (Table 1) showed that a slight reduction in the formation of tonsil caseum and in the ppb concentration of VSC and a slight increase in the level of tongue saburra.

**Table 1:** Results from the group that used the placebo solution.

Number of the volunteer	Frequency of caseum at every 04 weeks		Halitometry – VSC in PPB		Level of tongue saburra (from 0 to 5)	
	Initial	Final	Initial	Final	Initial	Final
1.	4	4	171	180	2	2
2.	1	0	151	26	2	2
3.	4	4	102	131	1	2
4.	1	10	54	35	0	1
5.	2	4	54	18	1	1
6.	4	4	155	79	1	1
7.	3	2	182	332	3	3
8.	4	3	151	20	2	1
9.	1	0	95	308	1	2
10.	2	2	156	127	3	1
11.	2	0	35	20	1	2
12.	4	1	514	49	2	1
13.	2	0	169	63	1	3
14.	4	1	84	75	1	1
15.	3	1	50	41	1	1
16.	2	0	46	39	1	1
17.	4	2	56	183	1	3
18.	8	1	180	116	1	1
19.	2	0	58	364	3	2
20.	1	1	123	96	1	1
21.	4	3	37	40	1	1
22.	3	3	72	76	1	2
23.	6	6	221	220	1	1
24.	4	0	46	59	3	1
25.	2	1	29	55	1	1
MEAN	3,08	2,12	119,64	110,08	1,44	1,52

For the t-Student test, carried out in the initial and final variables, to check the statistical significance of the group that used the placebo solution (Table 2), there was no significance among the samples.

**Table 2:** T-Student test carried out in the INITIAL and FINAL variables to check the statistical significance of the group that used the placebo solution.

Variable	t-Student	Significance
Frequency of caseum at every 04 weeks	1,78	Not significant, equal samples, alpha > 0.05.
Halitometry VSC in ppb	0,35	Not significant, equal samples, alpha > 0.05.
Intensity of tongue saburra (from 0 to 5)	0,40	Not significant, equal samples, alpha > 0.05.

As we checked the statistical correlation among the variables from the group that used the placebo solution (Table 3) we did not see any correlation among them.

**Table 3:** Pearson's correlation test applied to the variables after calculating the INITIAL data minus the FINAL at the end of treatment for the group that used the placebo solution.

Variables	r	Correlation
Caseum frequency at every 04 weeks X Halitometry – VSC ppb	0,0695	There is no correlation among the variables
Caseum frequency at every 04 weeks X Intensity of tongue saburra (from 0 to 5)	0,2155	There is no correlation among the variables
Halitometry – VSC ppb X Intensity of tongue saburra (from 0 to 5)	0,1815	There is no correlation among the variables

For the mean values of the results from the group that used the mouthwash (Table 4), there was a significant reduction in the tonsils caseum in the ppb concentration of VSCs and in the level of tongue saburra.

**Table 4:** Results from the group that used the mouthwash

Number of the volunteer	Frequency of caseum at every 04 weeks		Halitometry – VSC in PPB		Level of tongue saburra (from 0 to 5)	
	Initial	Final	Initial	Final	Initial	Final
26.	2	0	329	40	3	2
27.	3	1	54	58	1	1
28.	2	0	94	29	1	0
29.	2	0	296	62	3	1
30.	3	0	483	10	3	1
31.	2	0	35	15	1	1
32.	10	4	65	43	0	1
33.	4	2	414	133	4	2
34.	2	0	158	50	2	1
35.	12	2	212	48	1	1
36.	4	2	57	18	1	1
37.	8	2	139	53	2	1
38.	30	4	194	38	1	1
39.	2	1	55	20	3	1
40.	2	0	33	24	1	0
41.	8	1	55	42	1	1
42.	4	1	75	81	1	1
43.	2	0	18	75	2	2
44.	2	0	327	38	3	1
45.	3	0	40	17	1	1
46.	3	1	96	10	1	0
47.	2	1	75	08	1	1
48.	2	0	78	30	1	1
49.	2	1	65	19	1	1
50.	3	0	92	43	2	1
MEAN	4,76	0,92	141,56	40,16	1,64	1

For the t-Student test carried out in the initial and final variables, in order to check the statistical significance of the group that used the mouthwash (Table 5), there was statistical significance among the samples at the level of 1% (alpha = 0.01) in all the variables.

**Table 5:** T-Student test carried out in the INITIAL and FINAL variables to check the statistical significance of the group that used the mouthwash.

Variables	t-Student	Significance
Caseum frequency at every 04 weeks	3,78	Significante ao nível de 1% (alfa = 0,01).
VSC ppb from the halitometry	4,09	Significante ao nível de 1% (alfa = 0,01).
Intensity of tongue saburra (from 0 to 5)	3,72	Significante ao nível de 1% (alfa = 0,01).

As we checked the statistical correlation among the variables (Table 6) from the group that used the mouthwash, there was a correlation at the level of 1% (alpha = 0.01), for the variables Halitometry – ppb of VSCs X level of tongue saburra (from 0 to 5) (r = 0.6535).

**Table 6:** Pearson's correlation test applied to the variables after calculating the INITIAL data minus the FINAL from the treatment of the group that used the mouthwash.

Variables	r	Correlation
Caseum frequency at every 04 weeks	0,0847	There is no correlation among the variables
VSC ppb from the halitometry	-0,2712	There is no correlation among the variables
Intensity of tongue saburra (from 0 to 5)	0,6535	There is correlation at the level of 1% (Alpha = 0.01).

## DISCUSSION

For more than eight years treating patients with bad breath, we have seen many different methods and products have been used to reduce or even interrupt the formation of tonsil caseum, which is a problem that affects a significant portion of the population, especially those with halitosis.

As we surveyed the literature looking for conservative treatment options for pharyngeal tonsil caseum, we noticed that there is no non-invasive and low cost treatment method available today^4^^,^^12^. Laser cryptolysis is the only conservative method so far that offers good results, however it is costly and, depending on the technique employed – by volatilization or coagulation, it is painful in the post-op and often times requires many procedures.

Thus, based on the assumption that pharyngeal tonsil caseum etiology and mechanism of formation are the same as those of tongue saburra^1^, a hypothesis was formed that some substances that have proven effect on controlling tongue saburra could also act on tonsil caseum. Tests with different concentrations and formulations were carried out and some substances indeed had a positive effect in reducing caseum. When it worked, the substance was patented and, together with the State University of Campinas, the mouthwash was developed within excellence technical and scientific parameters. After that we followed it with a long stage of clinical tests in order to reach the formulation that would yield the best clinical results.

The products used for gargling and mouth washing usually have alcohol in their formulation, and this dries and dehydrates the oral mucosa, increasing cell shedding and, consequently, increasing tongue saburra and tonsil caseum. It happens because of proteolytic bacteria that degrade these scaled cells, which are protein remains that cause VSCs1 in this process. Thus, an important characteristic of this mouthwash we developed is that is does not have alcohol in its formula.

Another important characteristic of this mouthwash is the presence of oxygenators (one or more associated), which will act inhibiting the formation of caseum because they periodically oxygenate the tonsil crypts, making them less prone to anaerobic bacterial colonizing, and antimicrobial agents (one or more in association), in order to reduce the bacteria population present in the caseum forming sites. There are many studies showing that Hydrogen Peroxide^1^^,^^18^^,^^19^, Sodium Perborate^20^ and Cetylpyridinium Chloride^1^^,^^21^, ^22^, ^23^, ^24^ – active components used in the mouthwash formula, are a safe and efficient alternative in the treatment and prevention of gingivitis; and Cetylpyridinium Chloride is an effective anti-septic agent for oropharyngeal disorders^25^. Therefore, this new mouthwash could be recommended for patients with pharyngeal tonsil caseum, and also aid in the treatment of halitosis, because its components act on 3 of the main causes of oral-born halitosis: tongue saburra, gingivitis and tonsil caseum.

One factor that must be analyzed in the results obtained by the placebo solution in reducing tonsil caseum is probably due to the liquid turbulence caused by gargling twice a day. One hypothesis to be investigated for these results is that, those crypts more anatomically accessible by gargling may respond positively to the reduction in the formation of tonsil caseum, probably due to the cleaning action of gargling itself with the placebo solution inside the crypts.

## CONCLUSIONS

For the results presented by the group that used the placebo solution, there was neither statistically significant result nor variable correlation.

For the group that used the mouthwash solution, all results were statistically significant, and there was a correlation between VSC ppb – halitometry x level of tongue saburra, at the level of 1% (alpha = 0.01).

The mouthwash, when compared to placebo, yielded better results in all items analyzed, proving to be a feasible conservative alternative to treat caseous chronic tonsillitis, being also efficient in reducing VSC and tongue saburra.
